# Frequency of imaging phenotypes of pulmonary interstitial fibrosis

**DOI:** 10.4102/sajr.v29i1.3098

**Published:** 2025-05-09

**Authors:** Wallace T. Miller, Scott Simpson, Shweta Sood, Michelle Hershman, Cheilonda R. Johnson, James E. Schmitt, Karen C. Patterson

**Affiliations:** 1 Department of Radiology, Faculty of Medicine, University of Botswana, Gaborone, Botswana; 2 Department of Radiology, Faculty of Medicine, University of Pennsylvania, Philadelphia, United States; 3 Department of Internal Medicine, Faculty of Medicine, University of Pennsylvania, Philadelphia, United States; 4 Department of Internal Medicine, Faculty of Medicine, University of Sussex, Sussex, United Kingdom

**Keywords:** lung diseases, interstitial, pulmonary fibrosis, idiopathic pulmonary fibrosis, connective tissue diseases, sarcoidosis, asbestosis, alveolitis, extrinsic allergic, silicosis

## Abstract

**Background:**

Evaluation of diffuse interstitial lung disease (ILD) in thoracic imaging is complicated. Radiologists often use a pattern approach to interpretation; however, they are rarely aware of the statistical frequency of disease presentation.

**Objectives:**

To evaluate the relative frequency of causes of fibrotic ILD as a function of imaging patterns.

**Method:**

A CT database of 396 cases of fibrotic ILD was amassed from an institutional diffuse lung disease registry and retrospective search of medical records. Three radiologists and one pulmonologist independently and blindly reviewed the CT scans for the distribution of fibrosis, predominant feature and non-pulmonary findings.

**Results:**

Peripheral fibrosis was most common (291/396, 73.5%), usually caused by idiopathic pulmonary fibrosis (IPF) and connective tissue diseases-related interstitial lung disease (CTD-ILD) but occasionally by hypersensitivity pneumonitis (HP), idiopathic nonspecific interstitial pneumonia (iNSIP) and asbestosis. Peripheral fibrosis with honeycombing was usually IPF and without honeycombing, was usually CTD-ILD. Peripheral fibrosis with pleural plaques was always asbestosis. Peripheral fibrosis with oesophageal dilatation was usually connective tissue diseases. Consolidative-like peripheral fibrosis was CTD-ILD. Axial fibrosis (61/396, 15.4%) was usually sarcoidosis, HP, CTD-ILD or silicosis. Axial fibrosis with predominantly consolidative-like fibrosis, honeycombing, or reticulation was usually sarcoidosis. Axial fibrosis predominated by ground glass opacity was usually HP or CTD-ILD. Lymph node calcification or short axis > 17 mm increased the probability that axial fibrosis was due to sarcoidosis. The non-specific fibrosis phenotype was uncommon (44/396, 11.1%), usually CTD-ILD (25/44, 57%) but also HP, IPF, iNSIP or asbestosis.

**Conclusion:**

Patterns of lung fibrosis provide guidelines to identify the cause.

**Contribution:**

A flow diagram that predicts the relative frequency of the causes of 10 patterns of ILD.

## Introduction

Chronic fibrotic interstitial lung diseases (ILD) are important causes of respiratory impairment, including idiopathic pulmonary fibrosis-usual interstitial pneumonitis (IPF-UIP), connective tissue diseases-related interstitial lung disease (CTD-ILD), sarcoidosis, hypersensitivity pneumonitis (HP), idiopathic nonspecific interstitial pneumonia (iNSIP) and pneumoconiosis.^1,2,3^ The typical imaging appearance of IPF is characterised by fibrotic changes in the basilar and subpleural lungs.^4,5^ This pattern of fibrotic ILD has been most commonly studied. However, the frequency and diagnostic significance of fibrosis that is predominantly centred around the bronchovascular bundles (axial fibrosis) has not been evaluated, outside of the evaluation of individual lung diseases.

A multidisciplinary evaluation of the clinical, imaging and pathologic data is the most accurate means of diagnosing ILD.^6^ Because of the morbidity and mortality associated with surgical lung biopsy, biopsy is increasingly uncommon; and ILD diagnoses are frequently based on a combination of clinical, serologic and radiographic data.^4,6^

This study was designed to determine the relative frequency of the causes of fibrotic ILD based on imaging patterns and to determine which imaging features were most predictive of individual causes.

## Research methods and design

### Identification of cases

Cases of fibrotic ILD were acquired from two sources: (1) institutional ILD registry and (2) search of medical records for uncommon diseases. The University of Pennsylvania Medical Center began compiling a diffuse lung disease registry in January 2013. To be registered, cases were evaluated by two pulmonologists, a thoracic radiologist and a pulmonary pathologist who, in concert, classified the diagnosis by cause and confidence. Moderate and high confidence diagnosis cases were included in this study.

To capture cases not enrolled in the ILD database, our radiology information system (RIS) was searched over two time-intervals for five thoracic CT exam codes (Nuance mPower Clinical Analytics, Nuance Communications, Inc., Burlington, Massachusetts). The years from 2013 to 2018 were searched for exams with the following text: ‘asbestosis’, ‘desquamative interstitial pneumonia’, ‘DIP’, ‘hypersensitivity pneumonitis’, ‘Langerhans cell histiocytosis’, ‘LCH’, ‘miliary TB’, ‘miliary tuberculosis’, ‘respiratory bronchiolitis’, ‘RBILD’, ‘sarcoid’, ‘sarcoidosis’ and ‘silicosis’. The time frame for this search matched the ILD registry to maintain the relative frequency of diseases. The RIS was also searched over the years 2001–2018 for five thoracic CT exam codes and the search terms: ‘chronic beryllium disease’, ‘CBD’, ‘lymphocytic interstitial pneumonia’, ‘LIP’, ‘massive fibrosis’ and ‘talcosis’. The entire RIS was searched for these rare diseases in order to identify as many cases as possible.

Computed tomography reports from the medical record search were reviewed and cases were separated into those likely or unlikely to meet criteria for a diagnosis of ILD. Medical records from our institution of the likely cases were independently reviewed by a radiologist and a pulmonologist to determine if the case met criteria for a diagnosis of an ILD (Online Appendix 1).^4,5,7,8,9,10,11,12,13,14,15,16,17,18^ Both reviewers had to agree on the diagnosis for study inclusion.

Computed tomography features associated with fibrosis include honeycombing, reticulation, traction bronchiectasis and architectural distortion.^19,20,21,22,23^ Ground glass opacity (GGO) and consolidation are non-specific findings that frequently indicate alveolar filling. However, when seen in association with traction bronchiectasis or architectural distortion, GGO and consolidation-like opacities will usually indicate underlying microscopic and macroscopic fibrosis.^19,20,21,22,23^ This paper labels fibrosis with opacification that obscures the underlying interstitial markings, ‘consolidative-like fibrosis’.

**FIGURE 1:**
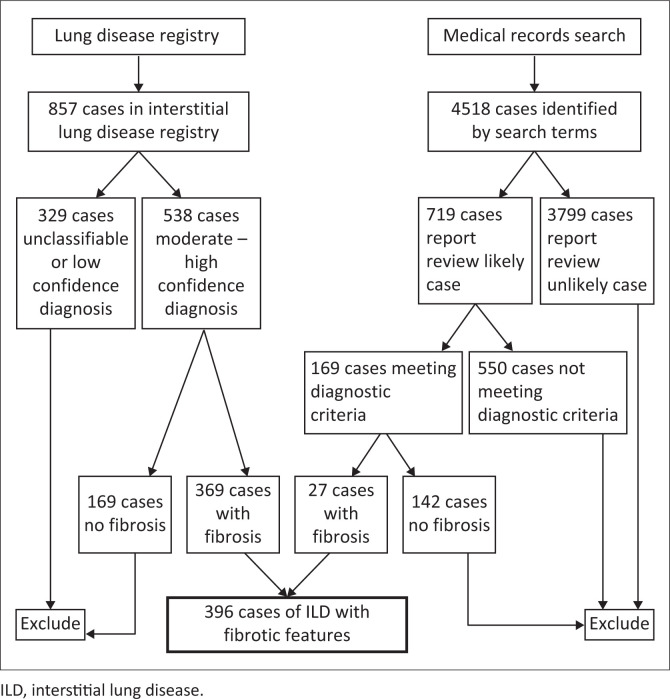
Sources of interstitial lung disease cases.

One radiologist (WM) reviewed the entire database for the presence of fibrotic findings and separated cases into those that contained at least one of the imaging findings of fibrosis and excluded those without fibrotic features. Figure 1 shows the numbers of cases in the database from each source.

### Image evaluation

Cases were anonymised, randomised, and blindly and independently reviewed by three radiologists and one pulmonologist. Radiologist 1, the creator of the database, evaluated the images 4 months after the database construction. Reviewers were blinded to clinical information, except for subject age and sex. Images were viewed on each reader’s personal computer using a DICOM imaging database (Horos, 2019 Horos project, https://horosproject.org/), that allows for scrollable images, window/level conversion and coronal and sagittal reconstructions.

**FIGURE 2:**
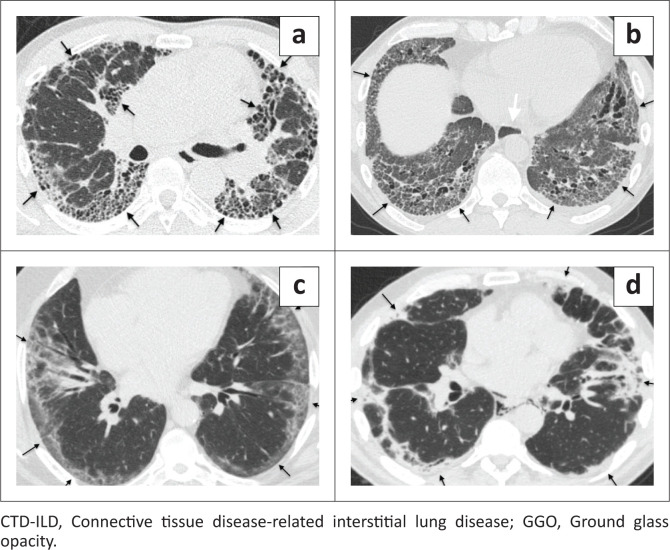
Peripheral fibrosis phenotype. (a–d) These four cases demonstrate fibrosis predominating in the subpleural lung, defining the peripheral fibrosis pattern. Features of this fibrosis pattern can include honeycombing, reticulation, GGO and solid scar. (a) 66-year-old man with IPF and peripheral fibrosis pattern with predominant honeycombing (black arrows). (b) A 57-year-old man with scleroderma and reticulation-predominant peripheral fibrosis pattern (black arrows). Also, the dilated oesophagus (white arrow) is a clue to the diagnosis of a CTD-ILD. (c) A 59-year-old man with hypersensitivity pneumonitis and peripheral fibrosis pattern predominated by GGO (black arrows). (d) A 61-year-old man with dermatomyositis and peripheral fibrosis pattern characterised by macroscopic regions of solid scar appearing as uniform high attenuation that obscures underlying interstitial markings and causes architectural distortion (black arrows). This sub-phenotype is highly specific for CTD-ILD.

**FIGURE 3:**
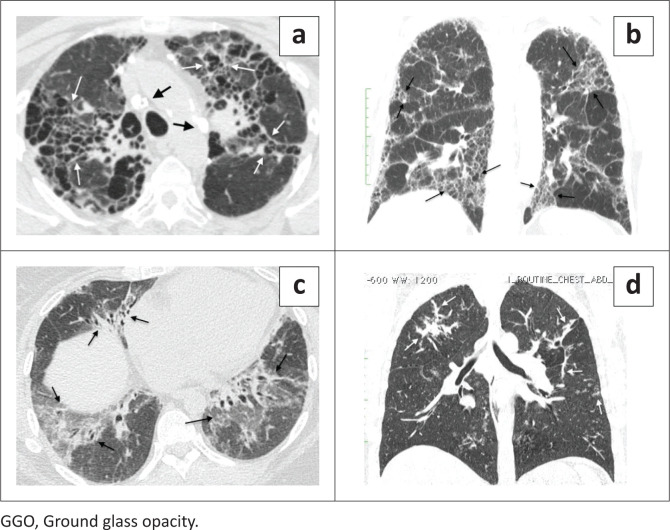
Axial fibrosis phenotype. (a–d) These four cases all show fibrosis predominating around the bronchovascular bundles, the axial fibrosis phenotype. However, the predominant feature of the fibrosis differs in each case. (a) 65-year-old woman with sarcoidosis and an axial fibrosis phenotype predominated by honeycombing (white arrows). Note the calcified lymph nodes (black arrows) is a clue to the diagnosis of a sarcoidosis. (b) A 58-year-old male farmer with hypersensitivity pneumonitis and an axial fibrosis pattern with reticulation as the predominant finding (black arrows). (c) 60-year-old woman with antisynthetase syndrome and axial fibrosis pattern predominated by GGO (black arrows). (d) 62-year-old woman with sarcoidosis and an axial fibrosis pattern characterised by bandlike fibrosis (white arrows).

**FIGURE 4:**
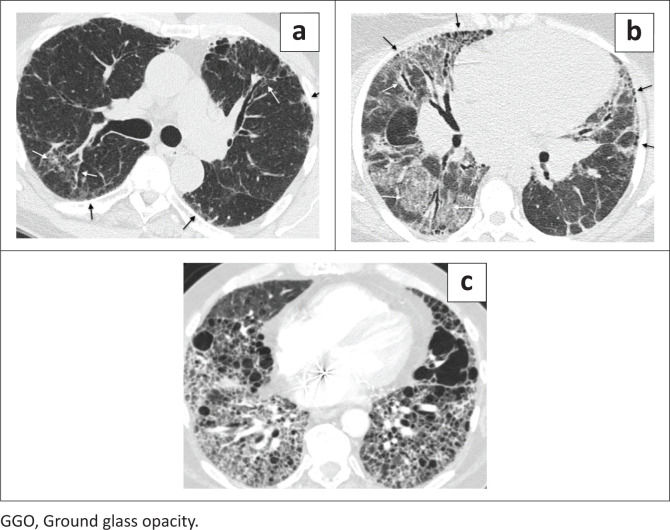
Nonspecific fibrosis phenotype. These images show areas of fibrosis involving both the subpleural lung (black arrows) and peribronchovascular interstitium (white arrows) in approximately equal amounts. In some cases, the disease involves the entire axial slice and in others it is heterogeneously scattered across the slice. This is the nonspecific fibrosis pattern. (a) A 68-year-old man with hypersensitivity pneumonitis and reticulation-predominant nonspecific fibrosis. (b) A 48 year-old woman with sarcoidosis and nonspecific fibrosis with predominant GGO. Note the hilar lymphadenopathy, which is a clue to the diagnosis of sarcoidosis. (c) A 70-year-old man with chronic beryllium disease and nonspecific fibrosis dominated by honeycombing.

Reviewers evaluated each case for the presence and predominant distribution of fibrosis: (1) subpleural lung (peripheral fibrosis phenotype, defined as predominating in the periphery) (Figure 2), (2) peribronchovascular lung (axial fibrosis phenotype, defined as surrounding the peribronchovascular interstitium) (Figure 3), (3) fibrosis in both the subpleural and peribronchovascular lung without predominance (nonspecific fibrosis phenotype) (Figure 4), or (4) not a fibrosis pattern. For each case, the reviewer also identified the dominant fibrosis feature: honeycombing, reticulation, GGO or consolidative-like fibrosis. The dominant feature was the one that was most prevalent among the images. Images were also evaluated for the presence of any honeycombing (when not the dominant feature), lymph node calcification, lymph node short axis > 17 mm, presence of pleural plaques and the presence of subjectively identified moderate or severe oesophageal dilatation.

In patients with axial fibrosis, the analysis showed that the dominant feature increased specificity. Therefore, ‘coarse axial fibrosis’ is defined as axial fibrosis with honeycombing or consolidative-like scar as the dominant feature and ‘fine axial fibrosis’ is defined as axial fibrosis with GGO as the dominant feature.

The conventional short axis diameter > 10 mm is known to have little specificity. Mild lymph node enlargement is found in many causes of ILD and may be a marker for active inflammation or disease.^24^ A short axis diameter > 17 mm was chosen by the principal investigator based on personal experience as a large enough diameter to exclude mildly enlarged lymph nodes because of non-specific inflammation or normal variation.

### Statistical evaluation

The Chi-square test or Fisher’s exact test was used to compare categorical data. All *p*-values reported are two-sided with a *p* < 0.05 considered statistically significant. To correct for multiple testing, the false discovery rate was applied. Inter-observer agreement was determined using Cohen’s kappa statistic. Sensitivity, specificity, accuracy, positive predictive value (PPV) and negative predictive value (NPV) for each ILD category were calculated for different imaging features.

### Ethical considerations

Ethical approval to conduct this study was obtained from the University of Pennsylvania, Institutional Review Board (reference no.: 820774). Informed consent was waived by the Institutional Review Board. The study is *Health Insurance Portability and Accountability Act* (HIPAA) compliant.

## Results

The causes of fibrotic ILD are listed in Table 1. Idiopathic pulmonary fibrosis and CTD-ILD accounted for 72.2% (286/396) of cases in our database. The next most common causes were sarcoidosis, HP and iNSIP, accounting for 20.5% (81/396) of cases. Only 7.3% (29/396) of cases were caused by another ILD. The orphan search process predominantly added pneumoconiosis: asbestosis, silicosis, chronic beryllium disease and talcosis.

**TABLE 1:** Causes of diffuse lung fibrosis.

Disease	Total database		Registry	
	*n*	%	*n*	%
Idiopathic pulmonary fibrosis	149	37.6	147	39.8
Connective tissue disease	137	34.6	137	37.1
Sarcoidosis	42	10.6	34	9.2
Hypersensitivity pneumonitis	28	7.1	28	7.6
Idiopathic NSIP	11	2.8	11	3.0
Asbestosis	9	2.3	1	0.3
Silicosis	6	1.5	0	0.0
Drug toxicity	3	0.8	3	0.8
Cryptogenic organising pneumonia	3	0.8	3	0.8
Aspiration	2	0.5	2	0.5
Chronic beryllium disease	2	0.5	0	0.0
Talcosis	1	0.3	0	0.0
LIP	1	0.3	1	0.3
Inflammatory bowel disease	1	0.3	1	0.3
Post ARDS	1	0.3	1	0.3

**Total**	**396**	**100.0**	**369**	**100.0**

ARDS, Adult respiratory distress syndrome; LIP, Lyphocytic interstitial pneumonia; NSIP, nonspecific interstitial pneumonia.

### Reader characteristics and inter-reader agreement

The radiologists had the following subspecialty experience in interpreting chest CT: reader 1 [R1] 28 years, reader 2 [R2] 5 years and reader 3 [R3] 1 year. The pulmonologist (R4) had 10 years of experience as a diffuse lung disease specialist. The readers had moderate (0.41–0.60) to substantial (0.61–0.80) agreement on the pattern of fibrosis.^25^ A ‘consensus pattern’ was defined as one where at least 3 of 4 readers agreed on the pattern and was established in 94.4% of cases (Table 2). Four readers agreed on the pattern in 59.0% (234/396) of cases, and an additional 3 of 4 readers agreed on the pattern in 26.3% (104/396) of cases. In 5.8% (23/396) of cases, 2 readers interpreted the case as non-specific and two readers interpreted it as peripheral fibrosis, while in 3.3% (13/396) of cases 2 readers interpreted the case as non-specific and two readers interpreted it as axial fibrosis. As these cases suggested peripheral or axial predominance, they were also added to the consensus pattern as peripheral and axial fibrosis, respectively. In 5.6% (22/396) of cases, there was no consensus on the pattern.

**TABLE 2:** Reader agreement on pattern and dominant finding.

Variable	4 agree		3 agree		2 × 2 agree		Total	
	*n*	%	*n*	%	*n*	%	*n*	%
**Pattern**								
Peripheral fibrosis	193	48.7	75	18.9	-	-	268	67.7
Axial scaring	33	8.3	15	3.8	-	-	48	12.1
Nonspecific scar	8	2.0	14	3.5	-	-	22	5.6
Peripheral scar/nonspecific scar	-	-	-	-	23	5.8	23	5.8
Axial scar/nonspecific scar	-	-	-	-	13	3.3	13	3.3
No consensus	-	-	-	-	-	-	22	5.6

**Total**	**234**	**59.0**	**104**	**26.3**	**36**	**9.1**	**396**	**100.0**

**Dominant feature**								
Reticulation	96	24.2	76	19.2	-	-	172	43.4
Honeycombing	25	6.3	26	6.6	-	-	51	12.9
Ground glass opacity	23	5.8	35	8.8	-	-	58	14.6
Uniform opacity	8	2.0	18	4.5	-	-	26	6.6
No consensus	-	-	-	-	-	-	89	22.5

**Total**	**152**	**38.4**	**155**	**39.1**	**-**	**-**	**396**	**100.0**

There was less agreement concerning the dominant feature, with lack of consensus in 22.5% (89/396) of cases. Cases often had multiple fibrotic features. If readers had been allowed to select more than one dominant feature, there may have been less disagreement. Disagreements were most common regarding whether a dominant feature represented reticulation or GGO, which were commonly present simultaneously.

### Peripheral interstitial fibrosis

Peripheral fibrosis was the most common consensus pattern of fibrosis in 73.5% (291/396) of cases (Table 3). Peripheral fibrosis was primarily caused by IPF (146/291, 50.2%) and CTD-ILD (106/291, 36.4%), together accounting for 86.6% of causes. Hypersensitivity pneumonitis, asbestosis and iNSIP combined, accounted for an additional 10.7% (31/291) of causes, leaving only 2.7% due to miscellaneous causes.

**TABLE 3:** Causes of diffuse lung fibrosis.

Disease	Total database	Fibrosis						Consensus (no)	
		Peripheral		Axial		Nonspecific			
	*n*	*n*	%†	*n*	%†	*n*	%†	*n*	%†
Idiopathic pulmonary fibrosis	149	146	98	0	0	2	1	1	1
Connective tissue disease	137	106	77	7	5	9	7	16	12
Sarcoidosis	42	1	2	36	86	4	10	1	2
Hypersensitivity pneumonitis	28	15	54	9	32	3	11	2	7
Idiopathic NSIP	11	7	64	3	27	1	9	1	9
Asbestosis	9	9	100	0	0	0	0	0	0
Silicosis	6	0	0	6	100	0	0	0	0
Drug toxicity	3	3	100	0	0	0	0	0	0
Cryptogenic organising pneumonia	3	2	67	1	33	0	0	1	33
Aspiration	2	1	50	0	0	1	50	0	0
Chronic beryllium disease	2	0	0	1	50	1	50	0	0
Talcosis	1	0	0	1	100	0	0	0	0
LIP	1	0	0	0	0	1	100	0	0
Inflammatory bowel disease	1	0	0	1	100	0	0	0	0
Post ARDS	1	1	100	0	0	0	0	0	0

**Total**	**396**	**291**	**-**	**65**	**-**	**22**	**-**	**22**	**-**

ARDS, Adult respiratory distress syndrome; LIP, Lyphocytic interstitial pneumonia; NSIP, nonspecific interstitial pneumonia.

† , Percent of each disease that presented with this pattern.

Peripheral interstitial fibrosis has been traditionally subdivided into those with honeycombing, the ‘interstitial pneumonia (UIP) pattern’, and those without. Just under half of the cases with peripheral fibrosis had a Usual interstitial pneumonia (UIP) pattern (114/256, 44.5%). A UIP pattern was most often associated with a diagnosis of IPF, which accounted for 73/114 (64.0%) of cases. (Figure 2a) Although the UIP pattern was strongly associated with IPF (*p* < 0.001), it had poor sensitivity (50.0%) and specificity (71.0%). Connective tissue diseases-related ILD also commonly caused a UIP pattern, accounting for 25.0% (28/114) of cases. Less common causes included asbestosis (4/114), iNSIP (3/114), HP (3/114), drug toxicity (2/114) and sarcoidosis (1/114), together accounting for 11.0% of UIP-pattern cases.

More than half of peripheral fibrosis cases had no honeycombing which was usually caused by CTD-ILD (72/142, 51.0%), IPF (48/142, 34.0%), and HP (10/142, 7.0%) (Figure 2b, Figure 2c, and Figure 2d). Uncommon causes included iNSIP (*n* = 4), asbestosis (*n* = 3), cryptogenic organising pneumonia (COP) (*n* = 2), drug toxicity (*n* = 1), aspiration (*n* = 1) and post adult respiratory distress syndrome (ARDS) (*n* = 1), which cumulatively caused 8.5% of cases.

Some radiographic findings increased diagnostic accuracy in patients with peripheral fibrosis (Table 4). Peripheral fibrosis with predominantly consolidative-like scar (Figure 2d) was associated with CTD-ILD (*p* = 0.01), with high specificity (99%) and PPV (86%). Peripheral fibrosis with a dilated oesophagus had a high specificity (97%) and PPV (78%) for CTD-ILD (*p* < 0.0001) (Figure 2b). Peripheral fibrosis with pleural plaques had a 100% specificity and 100% PPV for asbestosis (*p* < 0.0001) (Table 4).

**TABLE 4:** Performance of imaging features for the diagnosis of fibrotic lung diseases.

Imaging feature prediction for specific diagnosis	Sensitivity	Specificity	PPV	NPV	Accuracy	*p*
Peripheral fibrosis with honeycombing for IPF	50	71	64	58	60	0.0005
Peripheral fibrosis with oesophageal dilatation for CTD-ILD	17	97	78	67	68	< 0.0001
Consolidative-like peripheral fibrosis for CTD-ILD	6	99	86	65	65	0.0106*
Peripheral fibrosis with honeycombing for asbestosis	67	100	100	99	99	< 0.0001*
Coarse axial fibrosis for sarcoidosis	73	68	73	68	70	0.0036
Coarse axial fibrosis for sarcoidosis (silicosis excluded)	73	83	86	68	77	< 0.0001
Fine axial fibrosis for HP or CTD	44	88	58	81	76	0.0114*
Axial fibrosis with Abnormal LN for sarcoidosis	76	77	81	29	76	< 0.0001
Axial fibrosis with Abnormal LN for sarcoidosis (silicosis excluded)	76	95	96	29	83	< 0.0001

CTD, connective tissue disease; CTD-ILD, connective tissue disease-related interstitial lung disease; HP, hypersensitivity pneumonitis; IPF, idiopathic pulmonary fibrosis; LN, lymph node; NPV, negative predictive value; PPV, positive predictive value.

* , Fisher exact *p*-values.

### Axial interstitial fibrosis

The axial fibrosis phenotype accounted for 15.4% (61/396) of cases (Table 3), most commonly because of sarcoidosis (36/61, 59.0%), HP (8/61, 13.1%), pneumoconiosis (7/61, 11.5%) and CTD-ILD (6/61, 9.8%). Axial fibrotic pneumoconiosis included silicosis, coal workers’ pneumoconiosis, chronic beryllium disease and talcosis. Miscellaneous diseases accounted for only 6.6% (4/61) of axial fibrosis cases.

Sarcoidosis (21/42, 50.0%) and silicosis (5/6, 83.3%) typically cause axial fibrosis with the dominant finding of honeycombing or consolidative-like scar (‘coarse axial fibrosis’), which was rare in other cases of axial scarring (3/11, 27.2%) (Figure 3a and Figure 3d). Coarse axial fibrosis had 68% specificity and 73% PPV for sarcoidosis in this series which was enriched for pneumoconiosis cases. If silicosis is excluded on clinical grounds, these values increased to 83% specificity and 86% PPV (Table 4).

Lymph node calcification and/or enlargement with axial fibrosis were also associated with sarcoidosis and pneumoconiosis. Axial fibrosis with a lymph node abnormality had moderate sensitivity and specificity for a diagnosis of sarcoidosis, and if pneumoconiosis could be excluded on clinical grounds, measures increased to 76% sensitivity and 95% specificity (*p* < 0.0001) (Table 4 and Figure 3a).

The combination of fine axial fibrosis (only ground glass opacity with architectural distortion) with normal lymph nodes was insensitive (44%) but moderately specific for a diagnosis of either HP or CTD-ILD (Table 4 and Figure 3c).

### Nonspecific interstitial fibrosis and no consensus

Fibrosis that equally or nearly equally involved both the axial and peripheral interstitium was termed ‘nonspecific fibrosis’ and was seen in 22/396 (5.6%) of cases (Table 3 and Figure 4). Nonspecific fibrosis was most frequently caused by CTD-ILD (9/22, 41%). Nearly all diseases in the database occasionally caused nonspecific fibrosis with frequencies similar to the frequency of diseases in the database with some notable exceptions. Nonspecific fibrosis was caused by HP in 3/22 (14.0%) while representing only 2.8% of cases in the database. In contrast, IPF caused nonspecific fibrosis in 2/22 (9.0%) despite representing 37.6% of the total population.

**FIGURE 5:**
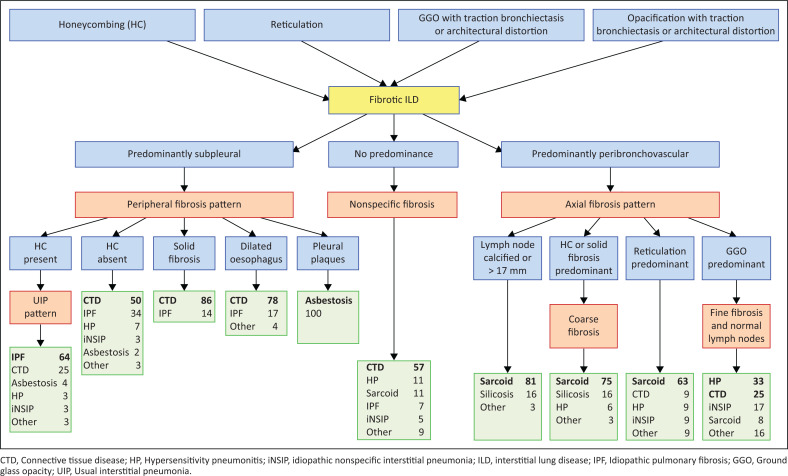
Interstitial fibrosis flow diagram. The diagram lists the discriminating imaging findings among causes of interstitial fibrosis. Patterns in orange, imaging findings in blue and the differential diagnosis in green. Numbers adjacent to diseases are the approximate frequency in percent of diseases causing the findings in the ILD database. Patterns were determined by 4 reader consensus.

No consensus on the pattern of fibrosis was most commonly caused by CTD-ILD in 16/22 (72.0%) of cases. Figure 5 is a flow diagram demonstrating the relative frequency of diagnoses observed in the cohort and the imaging features associated with them. There is considerable overlap in the imaging appearance of the five most common causes of interstitial fibrosis: IPF, CTD-ILD, sarcoidosis, HP and iNSIP. However, imaging features can indicate the most likely aetiology of interstitial fibrosis and provide a relative probability for each cause.

## Discussion

When imaging findings are non-specific, physicians are trained to develop a ‘differential diagnosis’ of possible causes that need to be considered in each individual patient’s case. Several factors will determine the relative likelihood of diagnoses with in a differential diagnosis. These include: (1) clinical presentation, (2) demographic features such as age, gender and ethnicity and (3) relative incidence of disease in the population. The incidence of fibrotic interstitial disease has recently been evaluated using US medical claims data and has shown that the majority of disease, approximately 75%, is because of IPF, with important smaller contributions due to sarcoidosis, connective tissue diseases (CTD) and inhalational exposures.^26,27^

In addition to these clinical factors, radiologists often use pattern recognition to help predict the cause of disease. Perhaps the greatest strength and utility of this study is that it provides a prediction of the *relative probability of diagnosis* based on *imaging features*, in addition to the clinical, demographic and epidemiological factors used by clinicians. Figure 5 provides the relative probability of diagnoses of 10 separate imaging patterns seen in this study population. The database utilised is one of the largest published collections of mixed cases of fibrotic lung disease. Nearly all cases were prospectively collected and adjudicated through a multidisciplinary review. Most cases that were added to the database from the medical record review were identified over the same time frame as the ILD registry. Therefore, the database, except for an oversampling of pneumoconiosis, should reflect the relative frequency of disease in a patient population from a Northeastern United States city. The results should align with other tertiary care referral centres but may overemphasise uncommon diseases when compared with primary care and community medical centres. Each institution will have a referral pattern of disease cases that is unique, however, this large database should reflect the overall relative frequencies of fibrotic ILD that are generally seen and their usual imaging appearances.

Peripheral fibrosis with honeycombing, the UIP pattern, has historically been linked with a pathologic diagnosis of UIP and the clinical diagnosis of IPF.^1,4,5^ While the current study results indicate that IPF is indeed the most common cause of radiographic UIP, it only accounts for 64% of cases of the UIP pattern, with the majority of other cases caused by CTD-ILD.

A peripheral fibrosis pattern without honeycombing has been associated with both UIP and NSIP histologically. The American Thoracic Society and Fleischer Society IPF criteria call this a ‘probable’ UIP pattern and studies suggest it will represent histologic UIP in 82% – 93% of cases of idiopathic lung disease.^4,5,28,29^ However, when this pattern is seen in association with CTD it often represents NSIP pathologically.^20,30^ The studied database suggests that the ‘probable’ UIP pattern of disease is most commonly caused by CTD-ILD (50%) followed by IPF (34%) when seen in the general population of patients with ILD. Since many of CTD-ILDs that cause this pattern will have underlying NSIP histologically, we believe the term ‘probable UIP pattern’ is problematic. Furthermore, the study data suggest that the traditional separation of peripheral fibrosis by the presence or absence of honeycombing, while useful demographically, has limited diagnostic utility in separating IPF, CTD-ILD, HP, asbestosis and iNSIP in individual cases. This study did not assess the extent of honeycombing, which has been shown to discriminate NSIP from UIP histologically by some authors.^31^

Certain additional imaging features in association with a peripheral fibrosis pattern may increase the probability of disease. Pleural plaques in association with a peripheral fibrosis phenotype was 100% specific for asbestosis in this study. A dilated oesophagus associated with the peripheral fibrosis phenotype had a 78% specificity for CTD-ILD. (Figure 2b) A peripheral fibrosis phenotype with consolidative-like fibrosis was 86% specific CTD-ILD (Figure 2d). Chung et al. identified some other pulmonary findings, ‘exuberant honeycombing’, the ‘straight edge sign’ and the ‘4 corner sign’ as markers that identify CTD-ILD from IPF that were not assessed in this study.^32^

The axial fibrosis phenotype has been less studied than peripheral fibrosis. This research found that recognition of this pattern can be diagnostically useful, especially if the dominant fibrotic features are considered. The coarse axial fibrosis phenotype (dominant feature either honeycombing or consolidative-like scaring) is typical of sarcoidosis and silicosis (Figure 3a and Figure 3d). Axial fibrosis with lymph node abnormality (calcification or short axis >17 mm) is associated with sarcoidosis and silicosis and when pneumoconiosis can be excluded on clinical grounds, will nearly always indicate sarcoidosis (Figure 3a).The fine axial fibrosis phenotype (GGO dominant feature) is rare in sarcoidosis and is usually because of other causes of axial fibrosis, HP, CTD-ILD and iNSIP (Figure 3c). Non-specific fibrosis was a rare pattern in this series and was occasionally caused by almost any ILD.

While a multi-disciplinary review is the gold standard approach to ILD diagnosis, an important limitation of this study is the lack of histologic confirmation of many cases of ILD, because of the practice patterns in our medical centre. A small fraction of cases were acquired by retrospective review of the medical records. An attempt was made to provide similar confidence of the diagnosis of these retrospectively acquired cases by requiring a radiologist and a pulmonologist to confirm the diagnoses, such as a multidisciplinary review. It is unclear what biases may have been introduced by this retrospective collection other than artifactually raising the frequency of these rare diseases, especially pneumoconiosis.

## Conclusion

The imaging evaluation of fibrotic ILDs is a complicated task that can be made more precise by a separation into three phenotypes, peripheral, axial and nonspecific fibrosis. There is considerable overlap in the imaging appearance of fibrotic lung diseases; however, the imaging phenotype is useful in determining the relative probability of causative diseases as demonstrated in Figure 5.

The presence or absence of honeycombing in patients with a peripheral fibrosis phenotype does not change the differential diagnosis but flips the relative probabilities of the two most common causes, IPF and CTD-ILD. Peripheral fibrosis with consolidative-like fibrosis will usually indicate a CTD-ILD. The presence of coarse or fine fibrosis in patients with an axial fibrosis phenotype flips the relative probabilities of the three most common causes, sarcoidosis, HP and CTD-ILD.

Some non-pulmonary findings also add specificity. The presence of pleural plaques or oesophageal dilatation in patients with the peripheral fibrosis phenotype will usually indicate a diagnosis of asbestosis and CTD-ILD, respectively. Lymph nodes larger than 17 mm and/or lymph node calcification in patients with axial fibrosis is highly predictive of sarcoidosis or silicosis.

### Key Points

1. The most common phenotype of fibrotic interstitial lung disease involves the subpleural (peripheral) lung and is usually due to IPF or a CTD-ILD.
2. Scarring predominantly around the bronchovascular bundles, the axial phenotype, is most often a feature of sarcoidosis followed by HP and CTD-ILD.
3. Some non-pulmonary findings including pleural plaques (asbestosis), moderate oesophageal dilatation (CTD-ILD) and lymph node calcification or enlargement (short axis diameter ≥ 17 mm) (sarcoidosis, silicosis), add specificity to the diagnosis of fibrosing interstitial lung diseases.
